# Quality of life of HIV-infected individuals: insights from a study of patients in Kermanshah, Iran

**DOI:** 10.1186/s12879-021-05908-z

**Published:** 2021-02-23

**Authors:** Nahid Khademi, Alireza Zanganeh, Shahram Saeidi, Raziyeh Teimouri, Mehdi Khezeli, Babak Jamshidi, Tan Yigitcanlar, Yahya Salimi, Ali Almasi, Kobra Gholami Kiaee

**Affiliations:** 1 Vice Chancellery for Disease Prevention and Control, Kermanshah University of Medical Sciences, Kermanshah, Iran; 2 Social Development and Health Promotion Research Center, Health Institute, Kermanshah University of Medical Sciences, Kermanshah, Iran; 3 UniSA Creative, University of South Australia, Adelaide, Australia; 4 School of Built Environment, Queensland University of Technology, Brisbane, Australia

**Keywords:** Quality of life, HIV patients, WHO model, Kermanshah, Iran

## Abstract

**Background:**

Quality of life (QOL) is one of the major factors to assessing the health and wellbeing of People living with HIV (PLWH). Likewise, improved QOL is among the prominent goals of patient treatment. This study was conducted to investigate the QOL of PLWH in Kermanshah, Iran.

**Methods:**

This cross-sectional study was conducted on 364 PLWH of Kermanshah between 2016 and 2017. Outpatients were selected as the sample through the convenience sampling method from HIV Positive Clients of Kermanshah Behavioral Diseases Counseling Center. The reasons for the selection of outpatients include: (a) some patients were substance users, homeless or did not have a fixed address to follow-up; (b) addresses and personal details that were registered on the first admission were incorrect or incomplete; (c) due to financial issues, some were forced to relocate frequently and were difficult to track; (d) some patients were convicts or prisoners, making it hard to find them after their release; (e) some of them were from other provinces, where managing access was not easy/possible. Data was collected using WHOQOL-HIV BREF questionnaire (Persian Version). Data also analyzed with STATA 14, and SPSS 23 using T-test and multiple regression.

**Results:**

This study showed that mean (SD) age of PLWH was 40.21 (10.45) years. Females had better QOL than males except for spirituality, religion and personal beliefs. The gender differences disappeared in multivariate results. A significant association was observed between education and the independence, environment, and spirituality domains of QOL. In addition, being married was correlated with overall QOL, psychological and social relationships domains of QOL of PLWH. Drug use was a behavioral factor with negative influence on the QOL.

**Conclusion:**

This study found that marital status and drug use were the main predictors of various domains of QOL. Drug use was a behavioral factor with a negative influence on the QOL. Hence, it is recommended that health professionals, planners, and policymakers take effective measures to improve the status quo.

## Background

HIV is one of the major health problems in the worldwide [1–8]. Most HIV infected people live in developing countries [9]. Although, governments and health organizations invest a lot to improve the health conditions of PLWH [1, 10–12], the QOL of PLWH still needs a special attention [12].

World Health Organization (WHO) defines QOL as “individual’s perception of their position in life in the context of the culture and value systems in which they live and in relation to their goals, expectations, standards and concerns” [13]. QOL is also used as one of the widely accepted theoretical frameworks to assessing the living conditions of patients [13]. It also is known as a key component of public health [14, 15], increasing the life span of patients [16, 17], affecting healthcare and daily activities, giving sense of goodness, and providing ability to resist various aspects of diseases [18, 19].

Improvement in antiretroviral therapy (ART) has led to increased survival in PLWH. Despite these improvements, HIV infection and its related problems still have a notable impact on health-related quality of life (HRQOL), even in people who are virally suppressed as a result of taking ART [4]. Specifically, individuals who have a history of injection drug use, or began ART with low CD4 cell counts have no reduction in mortality [20]. Supporting PLWH achieve positive effects in regard to their HRQOL requires understanding its determinants in this population. Studies have recognized a number of factors that are consistently associated with HRQOL among PLWH, including ageing, immunological status, the presence of symptoms, treatment adherence, depression, social support and employment [5, 6].

QOL is one of the key factors to evaluate the health status of PLWH, and its improvement is one of the important goals of treatment. Assessing the QOL can provide an accurate assessment of how patient life is affected by diseases and treatments [21]. More correctly, detecting the aspects of life, which are more affected by diseases are significant priorities for health professionals and policy makers. In recent years, the study of HRQOL, especially in chronic diseases, has become important. In Kermanshah, HIV infection has been recognized as a health problem [2–4, 22, 23]. Despite the efforts to contain the spread of the infection and prolong the lives of infected people by increasing access to antiretroviral drugs and improving clinical care, there has been less focus on patients’ QOL and associated factors [24]. The high burden of HIV in Kermanshah, together with increasing accessibility of HIV treatment and services, has increased the need for the assessment of QOL in PLWH. However, despite the growing trend of HIV infection in Kermanshah, no previous studies have been carried out about the effects of this disease on QOL of PLWH. Therefore, this study aimed to investigate the QOL of PLWH in Kermanshah, Iran.

## Methods

This is a cross-sectional study on HRQOL in a sample of HIV-infected people living in Kermanshah, Iran. Participants were identified via records in the counseling center, and then contacted to see if they were interested to participate. Participants were from the Kermanshah Behavioral Diseases Counseling Center (Kermanshah University of Medical Sciences and Health Services, Vice Chancellery for Disease Prevention and Control) between 2016 and 2017. Kermanshah Behavioral Diseases Counseling Center is the most prominent and only counseling center in the Western Iran. This center receipts HIV patients for diagnostic, counseling and treatment services. The sample consisted of available 364 PLWH selected through convenience sampling method. We used this method because: (a) some patients were substance users, homeless or did not have a fixed address to follow-up; (b) addresses and personal details that were registered on the first admission were incorrect or incomplete; (c) due to financial issues, some were forced to relocate frequently and were difficult to track; (d) some patients were convicts or prisoners, making it hard to find them after their release; (e) some of them were from other provinces, where managing access was not easy possible.

The patients who met the inclusion criteria, were invited to participate in the study. The inclusion criteria were certain diagnosis of HIV, patient readiness, voluntary participation and informed consent, having records at the counseling center, and the physical ability to answering the questionnaire. We considered patient readiness as a criterion for entering the study because: (a) some patients were unable to respond to the survey questionnaire due to different medical and psychological conditions, hence had to postpone the answering questionnaire to another time; (b) some patients were not ready to participate in the study because they were using drugs, so their response was not reliable. Hence, a counselor first assessed the patients to be provided with a questionnaire if they had readiness; otherwise, the completion of the questionnaire would have been postponed. Therefore, they were rescheduled for a later visit.

The consent to participate in the study was obtained in written consent form. The content of the consent form including explanation on aim of the study and confidentiality of information was read to the eligible patients that were interested to participate in the study, and informed consent was obtained from participants before the study. All procedures performed in the present study were in accordance with the ethical standards of the institutional and/or national research committee and also the 1964 Helsinki Declaration, and its later amendments or comparable ethical standards. An ethic approval was received from the ethics committee of Kermanshah University of Medical Sciences.

During the data collecting process, questionnaires were provided to the participants and the questioner was available to response any possible ambiguities. The data collected using self-administered questionnaire but for the illiterate participants was interviewer-administered. The person was given a choice for determined literacy status. The process of data gathering both with questionnaire and interview was conducted in counseling rooms at the clinic to protect patients’ privacy. Participants were assured that if they participate in the study or not, there would be no change in the services that are provided to them. There were no limitations in terms of the clinical stage of the disease. Demographic and basic data was gathered on gender, age, marital status, level of education (illiterate, primary education, the secondary education includes 7-9th grade, and high school includes 10-12th grade, University), self-rated health (SRH) (How is your health? Very Poor / Poor / Neither Poor nor Good / Good / Very Good), currently ill status (Do you consider yourself currently ill? Yes / No), HIV status (Asymptomatic / Symptomatic / AIDS converted), infected with HIV (Sex with a man / Sex with a woman / Injecting drugs / Blood products / Other), and years since diagnosis (see Appendix A).

The WHOQOL Questionnaire for HIV brief version (WHOQOL-HIV BREF) was used to assess patients’ QOL. The WHOQOL is a multidimensional, conceptualized, and generic questionnaire, consisted of 31-item rated on a five point Likert scale [25]. The WHOQOL-HIV BREF questionnaire consists of 31items and six domains: physical (Q3 + Q4 + Q14 + Q21), psycho-logical (Q6 + Q11 + Q15 + Q24 + Q31), level of independence (Q5 + Q22 + Q23 + Q20), social relations (Q27 + Q26 + Q25 + Q17), environment (Q12 + Q13 + Q16 + Q18 + Q19 + Q28 + Q29 + Q30) and spirituality, religion and personal beliefs (Q7 + Q8 + Q9 + Q10). Among the 31 items, 29 domain-specific items were used to measure individual QOL across six domains including physical, psychological, independence level, social relationships, environment and spirituality. Two items measure participants’ perception of general QOL and health status [26]. In most questions, options 1 and 5 represent the lowest and highest values respectively. However, in questions which a higher score did not mean better QOL, the responses were first inversely scored and then calculated. The score of each domain was calculated through adding total points of questions in each domain, dividing the total value by the number of questions, and finally multiplying the result by four. The score of each domain ranges between four and 20, according to which a score of four indicates the worst condition and a score of 20 represents the best in the domain [27]. This tool has been widely used in the world especially in Iran, and its validity and reliability have been confirmed [28, 29]. In this study, the overall QOL calculated based on all the 31items.

Data analysis was performed using STATA version 14 and SPSS version 23. The descriptive statistics and T-test were done in SPSS and regression analysis was done in STATA. Descriptive statistics were used to analyze demographic variables including gender, age, education, marital status, SRH, currently ill, HIV status, first positive test of HIV, and infected with HIV. Categorical variables were dummy coded for further analysis. Linear regression was done in enter method to assess the association of education level (Illiterate, primary education, secondary education, and university education), marital status (single, married, divorced and widow), HIV transmission (sex with a man, sex with a woman, injecting drugs, blood products and other), age, gender and health with each QOL domains. The enter method an appropriate analysis when dealing with a small set of predictors and when we do not know which independent variables will create the best prediction equation. Each predictor is assessed as though it were entered after all the other independent variables were entered, and assessed by what it offers to the prediction of the dependent variable that is different from the predictions offered by the other variables entered into the model [30]. Also, the variables for inclusion in the models were entered into the equation at the same time. We estimated 95% confidence intervals for each of the estimated regression coefficients.

## Results

Among 364 patients, 225 (61.81%) were male and 139 (38.19%) were female, and the mean and standard deviation of their age was 40.21(SD = 10.45) years. In addition, the mean and standard deviation of males and females ages were 40.91(SD = 10.47) and 39.07(SD = 10.35), respectively. Marital status of PLWH were 24.5% single, 47% married, 13.7% divorced, and 14% widowed. In addition, most of patients (37.9%) had secondary education and 26.4% primary education (Table 1).

**Table 1:** Demographic characteristics of PLWH in Kermanshah

Variables	Male	Female	Total
**Gender**	225(61.8)	139(38.2)	364(100)
**Age in years**			
Mean (SD)	40.91(10.47)	39.07(10.35)	40.21(10.45)
**Education level n(%)**			
Illiterate	9(4)	25(18)	34(9.3)
Primary education	53(23.6)	43(30.9)	96(26.4)
Secondary education	97(43.1)	41(29.5)	138(37.9)
University	66(29.3)	30(21.6)	96(26.4)
**Marital status n(%)**			
**Single**	**82(36.4)**	**7(5)**	**89(24.5)**
**Married**	**103(45.8)**	**71(51.1)**	**171(47)**
**Divorced**	**37(16.4)**	**13(9.4)**	**50(13.7)**
**Widow**	**3(1.3)**	**48(34.5)**	**51(14)**
**HIV status n(%)**			
vAsymptomatic	13(5.8)	28(20.1)	41(11.3)
Symptomatic	27(12)	19(13.7)	46(12.6)
AIDS	185(82.2)	92(66.2)	277(76.1)
**Infection period n(%)**			
> 5 years	37(16.4)	25(18)	62(17)
≤ 5 years	188(83.6)	114(82)	302(83)
**HIV transmission n(%)**			
Sex with a male or female	28(18.06)	127(81.9)	155(42.6)
Injecting drugs	164(99.4)	1(0.6)	165(45.3)
Other	33(75.0)	11(25.0)	44(12.1)

Among the participants, 39.3% were suffering from other illnesses in addition to HIV infection including Hepatitis C (the spread of Hepatitis C is probably because of intravenous drug use, blood transfusions, and unsafe medical practices, respectively), Tuberculosis (tuberculosis causes a lot of complications in PLWH which can occurred at all stages of the HIV-infection, and similarly HIV can increases the number of people infected with tuberculosis and transmission of tuberculosis in the community), and psychosocial illnesses (such as depression, mood disorder, and schizophrenia). The results revealed that the CDC classification in patients was as follows: 11.3% asymptomatic, 12.6% symptomatic, and 76.1% AIDS converted. Also, the most common ways of HIV transmission were injecting drugs (45.3%), and sex with a male or female (42.6%) (Table 1).

The mean score of patients’ QOL for the whole domains of the questionnaire was 12.19(2.44). Moreover, the level of independence had the lowest mean score (9.92(2.90)), whereas the domain of spirituality, religion and personal beliefs had the highest mean score (13.85(3.21)) (Table 2). Besides, the results of comparing male and female patients demonstrated that except for spirituality, religion and personal beliefs, females had better conditions than males in terms of all aspects of QOL (Table 2).

**Table 2:** Results of mean and standard deviation of QOL of PLWH

QOL domain	Gender			T-test	
	Male	Female	Total		
	Mean (SD)	Mean (SD)	Mean (SD)	t	*P*-value
**Physical**	12.64 (4.05)	14.42 (3.35)	13.32 (3.89)	4.538	0.000
**Psychological**	9.90 (3.40)	11.21 (3.29)	10.40 (3.42)	3.611	0.000
**Level of independence**	9.34 (3.03)	10.87 (2.38)	9.92 (2.90)	5.358	0.000
**Social relationships**	12.31 (3.46)	14.28 (2.70)	13.06 (3.33)	6.032	0.000
**Environment**	12.34 (1.87)	12.83 (1.86)	12.52 (1.88)	2.417	0.016
**Spirituality**	13.97 (3.14)	13.66 (3.33)	13.85 (3.21)	−0.894	0.372
**Overall QOL**	11.78(2.52)	12.87 (2.14)	12.19 (2.44)	4.354	0.000

### Regression analysis for QOL of PLWH

Multiple regression analysis was done with the mentioned significant variables and showed the following results: the first model for physical health variable was statistically significant (p < 0.001; R2 = 0.18) with the main predictors of injecting drugs and SRH. For example, with each one-unit increase in the SRH score, the physical domain score increased by 0.62 units (*p* < 0.001) (Table 3). The second model for psychological health was significant (p < 0.001; R2 = 0.12), in which the main predictors were marital status and injecting drugs. The mean score of psychological health was 1.2 units lower in those who infected by injecting drug compared to those who infected by sexual relationship (*p* = 0.03). In third model for level of independence, variables of primary education, marital status, injecting drugs and SRH were significant predictors (*p* < 0.001; R2 = 0.21). The mean score of level of independence domain was 1.18 units higher in married people compared to single people (*P* < 0.001). The main significant predictors of social relationships domain in fourth model were the marital status and injecting drugs (*p* < 0.001; R2 = 0.22). The fifth model was statistically significant for environment (p < 0.001; R2 = 0.11) with the main predictors of secondary education, university’s education and injecting drugs. The sixth model included Spirituality domain was statistically significant (*p* < 0.003; R2 = 0.03) with main predictors of primary education, and marital status. The final model for predictors of overall QOL was significant (p < 0.001; R2 = 0.20) in which the main predictors were marital status and, injecting drugs. For example, the mean score of overall QOL domain was 1 unit higher in married people compared to single people (*P* < 0.001). The effects of the other variables were not significant (Table 3).

**Table 3:** Regression coefficients of linear regression analyses for significant predictors of QOL and its domains in PLWH in Kermanshah

Models^a^	Variables^b^	Categories	Coefficient	Std. Error	t	***P***-value	R-squared	Adjusted R Square	***P***-value
**(1) Physical**	Education level	Illiterate	–	–	–	–	0.20	0.18	< 0.001
		Primary education	−1.19	.71	−1.67	0.09			
		Secondary education	.31	.72	0.44	0.66			
		University	.93	.74	1.26	0.21			
	Marital status	Single	–	–	–	–			
		Married	.25	.52	0.49	0.62			
		Divorced	−1.15	.65	−1.78	0.07			
		Widow	−.97	.80	− 1.21	0.22			
	***HIV transmission***	Sex with a male or female	–	–	–	–			
		***Injecting drugs***	***−2.67***	***.68***	***−3.93***	***< 0.001***			
		Other	−.75	.72	−1.04	.29			
	Age	**–**	−.03	.02	−1.45	0.14			
	***SRH***	***–***	***.62***	***.19***	***3.22***	***< 0.001***			
	Sex	–	.12	.65	.19	.84			
	Constant	–	13.86	1.46	9.48	***< 0.001***			
**(2) Psychological**	Education level	Illiterate	–	–	–	–	0.14	0.12	< 0.001
		Primary education	−.67	.65	−1.03	0.30			
		Secondary education	.07	.65	0.12	0.90			
		University	.43	.67	0.64	0.52			
	***Marital status***	Single	–	–	–	–			
		***Married***	***1.32***	***.47***	***2.78***	***< 0.001***			
		Divorced	−.48	.59	−0.81	0.41			
		Widow	−.06	.73	−0.09	0.92			
	***HIV transmission***	Sex with a male or female	–	–	–	–			
		***Injecting drugs***	***−1.2***	***.62***	***−2.07***	***0.03***			
		Other	.29	.66	0.45	0.65			
	Age	–	−.01	.02	−0.81	0.41			
	*SRH*	–	.32	.17	1.80	0.07			
	Sex	–	−.23	.60	−0.39	0.69			
	Constant	–	10.48	1.33	7.85	0.00			
**(3) Level of independence**	***Education level***	Illiterate	–	–	–	–	0.23	*0.21*	< 0.001
		***Primary education***	***−1.23***	***.52***	***−2.35***	***0.01***			
		Secondary education	−.37	.52	−0.70	0.48			
		University	.04	.54	0.09	0.92			
	***Marital status***	Single	–	–	–	–			
		***Married***	***1.18***	***.38***	***3.10***	***< 0.001***			
		Divorced	−.15	.47	−0.32	0.74			
		Widow	−.08	.58	− 0.14	0.89			
	***HIV transmission***	Sex with a male or female	–	–	–	–			
		***Injecting drugs***	***−2.09***	***.50***	***−4.18***	***< 0.001***			
		Other	−.21	.53	−0.40	0.69			
	Age	–	−.00	.01	−0.52	0.60			
	***SRH***	***–***	***.29***	***.14***	***2.04***	***0.04***			
	Sex	–	.13	.48	0.28	0.77			
	Constant	–	10.01	1.07	9.35	0.00			
**(4) Social relationships**	Education level	Illiterate	–	–	–	–	0.25	0.22	< 0.001
		Primary education	−.71	.59	−1.19	0.23			
		Secondary education	.04	.60	0.08	0.93			
		University	−.08	.61	−0.14	0.88			
	***Marital status***	Single	–	–	–	–			
		***Married***	***2.58***	***.43***	***5.98***	***< 0.001***			
		Divorced	.01	.54	0.03	0.97			
		***Widow***	***1.52***	***.66***	***2.29***	***0.02***			
	***HIV transmission***	Sex with a male or female	–	–	–	–			
		***Injecting drugs***	***−1.25***	***.56***	***−2.20***	***0.02***			
		Other	.16	.60	0.27	0.78			
	Age	–	−.01	.01	−0.62	0.53			
	*SRH*	***–***	.24	.16	1.51	0.13			
	Sex	–	−.34	.54	−0.63	0.52			
	Constant	–	12.60	1.21	10.35	0.00			
**(5) Environment**	***Education level***	Illiterate	–	–	–	–	0.14	0.11	< 0.001
		Primary education	.26	.36	0.74	0.45			
		***Secondary education***	***.81***	***.36***	***2.25***	***0.02***			
		***University***	***1.12***	***.37***	***3.03***	***< 0.001***			
	Marital status	Single	–	–	–	–			
		Married	.37	.26	1.44	0.15			
		Divorced	−.59	.32	−1.81	0.07			
		Widow	−.40	.40	−1.00	0.31			
	***HIV transmission***	Sex with a male or female	–	–	–	–			
		***Injecting drugs***	***−1.28***	***.34***	***−3.75***	***< 0.001***			
		Other	−.44	.36	−1.22	0.22			
	Age	–	.00	.01	0.44	0.65			
	*SRH*	***–***	.02	.09	0.21	0.83			
	Sex	–	.21	.33	0.66	0.51			
	Constant	–	11.82	.73	16.06	0.00			
**(6) Spirituality**	***Education level***	Illiterate	–	–	–	–	0.06	0.03	< 0.001
		***Primary education***	***−1.58***	***.64***	***−2.46***	***0.01***			
		Secondary education	−.98	.64	−1.53	0.12			
		University	−.65	.66	−0.98	0.32			
	***Marital status***	Single	–	–	–	–			
		Married	.57	.46	1.23	0.21			
		***Divorced***	***−1.35***	***.58***	***−2.31***	***0.02***			
		Widow	.01	.71	0.02	0.98			
	HIV transmission	Sex with a male or female	–	–	–	–			
		Injecting drugs	−.60	.61	−0.99	0.32			
		Other	−.04	.64	−0.06	0.94			
	Age	–	.00	.02	0.27	0.78			
	*SRH*	***–***	−.04	.17	−0.26	0.79			
	Sex	–	.91	.59	1.54	0.12			
	Constant	–	13.46	1.31	10.27	0.00			
**(7) Overall QOL**	Education level	Illiterate	–	–	–	–	0.22	0.20	< 0.001
		Primary education	−.82	.44	−1.84	0.06			
		Secondary education	−.01	.44	−0.02	0.98			
		University	.35	.46	0.76	0.44			
	***Marital status***	Single	–	–	–	–			
		***Married***	***1.00***	***.32***	***3.10***	***< 0.001***			
		Divorced	−.68	.40	−1.69	0.09			
		Widow	−.07	.49	−0.14	0.88			
	***HIV transmission***	Sex with a male or female	–	–	–	–			
		***Injecting drugs***	***−1.62***	***.42***	***−3.81***	***< 0.001***			
		Other	−.25	.45	−0.58	0.56			
	Age	–	−.00	.01	−0.58	0.56			
	SRH	**–**	.22	.12	1.89	0.05			
	Sex	–	.20	.41	0.49	0.62			
	Constant	–	12.00	.90	13.24	***< 0.001***			

^a^Variables education level (Illiterate, primary education, secondary education, and university education), marital status (single, married, divorced and widow), HIV transmission (sex with a man, sex with a woman, injecting drugs, blood products and other), age, gender and health included in the all models. ^b^significant variables in the all models

## Discussion

Our study showed that QOL in PLWH was lower among PLWH in other countries [31, 32]. In many studies, poor QOL was associated with a lower immune response, non-adherence, poor mental health, and greater disease severity [8, 33]. Therefore, educational and supportive interventions could be designed and implemented by health sector in Kermanshah to promote the QOL in PLWH. In addition, we found poorer QOL in the psychological and level of independence domains compared to other domains. In this regard, a study showed that low mean score of psychological and level of independence indicated the lower levels of social and structural support [34]. On the other hand, the finding indicated that spirituality, religion and personal beliefs had the highest score among domains, which was consistent with the findings of other studies conducted in Iran [29, 35]. The previous literature has reported a complex combination of psychological and social factors which also influenced their physical, mental and social conditions, directly and indirectly, affect their QOL [8].

The results showed that females had better conditions in QOL domains score than males except for spirituality, religion and personal beliefs (Table 2). A previous study in Iran concluded that women had a better QOL than men in terms of psychological status and spirituality [29]. On the other hand, several studies showed that females had significantly lower QOL compared with males [36–38]. In addition, Belak et al., showed that women had undesirable QOL in terms of physical and psychological activities, environment, independence, and spirituality [39]. Also, in studies conducted in western countries gender showed no major impact on QOL [36, 40]. However, economic and social inequality and discrimination against women can constraint their access to health care, treatment, and supportive services in settings with limited health care resources. These barriers could make women more vulnerable to the physical and psychological burden of HIV [36, 41]. In Kermanshah, the possible reason for males scoring lower QOL than females mostly the men were injection drug users. In addition, previous study showed that Kermanshah faces many challenges, including increasing poverty, high rate of unemployment for males, economic recession, the concentration of low-income and mostly immigrant households [4, 22, 42]. This situation has probably affected the QOL of males living with HIV in Kermanshah. Although In this study the gender difference in QOL was significant, the multivariate results showed that gender was no longer significant when the other variables were taken into account. As a result, the explanation for men having lower QOL could be that they mostly are injection drug users, and this can be supported by the fact that drug use is significant in some of the multivariate models, and gender wasn’t. So it can be said that other factors that correlate with the gender that explain the apparent differences in QOL between the genders.

A significant association was observed between education and the independence, environment, and spirituality domains in the present study. Also, the only category that was significantly different from illiterate was primary education, and the latter scored lower than the illiterate participants for 2 of the domain’s independence and Spirituality. Previous studies showed that people with higher education (e.g. those who finished University) were more likely to have a good general QOL as well as better QOL scores in the physical, environment, and independence domains compared to those with low education. Education potentially provides opportunities for employment and social support, and thus can contribute to a sense of better QOL [43, 44]. A previous study reported that low educational level contributed in spreading HIV in community and a social vulnerability of poor people especially in HIV-infected women [45]. Results of another study also showed that higher education was significantly associated with good QOL [46]. A higher educational level may promote awareness regarding the treatment, as well as better access to health services [47]. It reflects that education enhances problem-solving and active decision-making making the patient to cope with the dreaded disease better, both emotionally and problem focused [48]. Improving education levels potentially provides opportunities for employment and social support, and thus can contribute to a sense of better QOL. Also, may promote awareness regarding the treatment, as well as better access to health services.

Our result showed that being married compared to single person was correlated with overall QOL, psychological and social relationships of PLWH. This makes sense; as a married person would have more opportunities to socialize with at least one other person who could be their partner, also married persons have families that can be a source of social support. Previous of studies identified being married or in a stable relationship as associated to a better QOL [49, 50], and being unmarried was significantly associated to lower mental and physical dimensions of QOL [50]. In addition, HIV-positive married people have longer and healthier lives compared to other marriage groups, and marriage has a significant impact on normalizing life cultivating personal growth and fulfillment [51]. On the other hand, the findings showed that being married had negative affected in dominos independence level, environment and spirituality (Table 3). In another studies also emphasized being married and having further number of sex partners and sex acts have negative affected with QOL of PLWH [29, 52, 53]. It is likely that most infected women have contracted HIV through sexual contact with their husbands. Besides, HIV transmission through sexual intercourse was more prevalent in women than men were since the husbands of these women were infected with HIV due to intravenous drug injection or unsafe sexual contacts, thereby transferring it to their women. In addition, the study showed that among patients participating in this study, the percentage of single and divorced women was lower than the married and widowed women. This was probably affected by the cultural structures of the Iranian society in which families have more control over women. On the other hand, the percentages of married and widow females’ patients in total women were higher in comparison with males. As has been noted in other studies, some HIV-infected women are those who are forced into prostitution for a living, and the poor economic situation of widows is reported as a major factor in this regard [54, 55]. However, as expressed in other studies, HIV/AIDS infected men with injecting drug addiction are probably accounted factor for HIV spread in women [56, 57].

This study showed that mean (SD) age of patients was 40.21 (10.45) years. Different results were found in another Iranian study performed with PLWH, they were lower than our results (the mean ± SD = 34.3 ± 7.5) [29], but the mean age of the study in Ibadan, Nigeria was 41.3 ± 10 years that were higher than Iranian results. Also, our study demonstrated that the mean age of HIV-infected males was higher than females, which was consistent with the results of other study [2]. On the other hand, previous studies in Kermanshah over 1996–2014 indicated that the mean age of patients was decreasing [2, 3, 58]. As compared with other studies in Kermanshah, in the case of access to anti-HIV drugs, the mean age of patients increases [59]. The results suggest that the patients who have received services from the Center for Communicable Diseases in Kermanshah has longer lifespan than other patients. Previous research showed that HIV-care, ART and health improvement were associated with an increase in mean age [59]. As mentioned in other studies, the observation of a mean older age in PLWH is proof that ART works [60].

### Limitations

We have several important limitations in this study. Firstly, it is difficult to determine the causation relationship between factors investigated in a cross-sectional study. Second, it is difficult to obtain a truly representative sample of a community HIV population. Third, using self-report questionnaires, information bias about HIV behaviors is possible.

## Conclusion

The independent variables included in regression models of this study explained variances of QOL domains from 3% (for the spirituality domain) to 22% (for social relationships domain). This study found that marital status and drug use were the main predictors of various domains of QOL. Drug use was a behavioral factor with a negative influence on the QOL. Hence, the most immediate course of action to try to improve the lives of the PLWH in this study is to help them with their drug use. Those who reported becoming infected via drug use (which isn’t the same as currently using drugs, necessarily) have a lower QOL than those infected via sex with a man. Hence, it is recommended that health professionals, planners, and policymakers take effective measures to improve the status quo. In view of these findings, being married had a positive impact on all aspects of QOL in HIV patients except environment and spirituality domains.

## Data Availability

The data sets used and analyzed in this study are available from the corresponding author on reasonable request.

## Appendix

### WHOQOL-HIV BREF

Before you begin, we would like to ask you to answer a few general questions about yourself: by circling the correct answer or by filling in the space provided.

What is your **gender**? Male / Female.

How old are you? _________________ (age in years).

What is the highest **education** you received? Illiterate/ primary education/ Secondary education/ University.

What is your **marital status**? Single / being married / Temporary marriage / Separated / Divorced / Widowed.

How is your **health**? Very Poor / Poor / Neither Poor nor Good / Good / Very Good.

Do you consider yourself currently ill? Yes / No.

If there is something wrong with you, what do you think it is? _________________.

**Table:**

***Please respond to the following questions if they are applicable to you:*** What is your **HIV status**? Asymptomatic / Symptomatic / AIDS converted In what year did you first **test positive** for HIV? _________________ In what year do you think you were infected? _________________ How do you believe you were **infected with HIV**? (circle one only): Sex with a man / Sex with a woman / Injecting drugs / Blood products / Other (specify)_________________	

***Instructions.***

This assessment asks how you feel about your quality of life, health, or other areas of your life. **Please answer all the questions.** If you are unsure about which response to give to a question, **please choose the one** that appears most appropriate. This can often be your first response. Please keep in mind your standards, hopes, pleasures and concerns. We ask that you think about your life **in the last two weeks.** For example, thinking about the last two weeks, a question might ask:

**Table:**

		Not at all	A little	A moderate amount	Very much	Extremely
11(F5.3)	How well are you able to concentrate?					

**You should circle the number that best fits how well you are to concentrate over the last two weeks. So you would circle the number 4 if you were able to concentrate very much. You would circle number 1 if you were not able to concentrate at all in the last two weeks.**

**Please read each question, assess your feelings, and circle the number on the scale for each question that gives the best answer for you.**

**Table:**

		Very poor	Poor	Neither poor nor good	Good	Very good
1(G1)	How would you rate your quality of life?

**Table:**

		Very dissatisfied	Dissatisfied	Neither satisfied nor dissatisfied	Satisfied	Very satisfied
2 (G4)	How satisfied are you with your health?					

The following questions ask about **how much** you have experienced certain things in the last two weeks.

**Table:**

		Not at all	A little	A moderate amount	Very much	An extreme amount
3 (F1.4)	To what extent do you feel that physical pain prevents you from doing what you need to do?					
4(F50.1)	How much are you bothered by any physical problems related to your HIV infection?					
5(F11.3)	How much do you need any medical treatment to function in your daily life?					
6 (F4.1)	How much do you enjoy life?					
7(F24.2)	To what extent do you feel your life to be meaningful?					
8(F52.2)	To what extent are you bothered by people blaming you for your HIV status					
9(F53.4)	How much do you fear the future?					
10(F54.1)	How much do you worry about death?

**Table:**

		Not at all	A little	A moderate amount	Very much	Extremely
11 (F5.3)	How well are you able to concentrate?					
12 (F16.1	How safe do you feel in your daily life?					
13 (F22.1	How healthy is your physical environment?					

The following questions ask about **how completely** you experience or were able to do certain things in the last two weeks.

**Table:**

		Not at all	A little	Moderately	Mostly	Completely
14 (F2.1)	Do you have enough energy for everyday life?					
15 (F7.1)	Are you able to accept your bodily appearance?					
16 (F18.1)	Have you enough money to meet your needs?					
17 (F51.1)	To what extent do you feel accepted by the people you know?					
18 (F20.1)	How available to you is the information that you need in your day-to-day life?					
19 (F21.1)	To what extent do you have the opportunity for leisure activities?

**Table:**

		Very poor	Poor	Neither poor nor good	Good	Very good
20 (F9. 1)	How well are you able to get around?					

The following questions ask you how **good or satisfied** you have felt about various aspects of your life over the last two weeks.

**Table:**

		Very dissatisfied	Dissatisfied	Neither satisfied nor dissatisfied	Satisfied	Very satisfied
21(F3.3)	How satisfied are you with your sleep?					
22(F10.3)	How satisfied are you with your ability to perform your daily living activities?					
23(F12.4)	How satisfied are you with your capacity for work?					
24 (F6.3)	How satisfied are you with yourself?					
25(F13.3)	How satisfied are you with your personal relationships?					
26(F15.3)	How satisfied are you with your sex life?					
27(F14.4)	How satisfied are you with the support you get from your friends?					
28(F17.3)	How satisfied are you with the conditions of your living place?					
29(F19.3)	How satisfied are you with your access to health services?					
30(F23.3)	How satisfied are you with your transport?					

The following question refers to **how often** you have felt or experienced certain things in the last two weeks.

**Table:**

		Never	Seldom	Quite often	Very often	Always
31(F8.1)	How often do you have negative feelings such as blue mood, despair, anxiety, depression?					

## Abbreviations

QOL: Quality of life
WHO: World Health Organization
SRH: Self-rated health
